# Editorial

**Published:** 1902-02-15

**Authors:** 


### EDITORIAL.

The Ninth Annual Meeting of the Institute of Dental Pedagogics was held at Pittsburg, December 31st and January 1st and 2nd. It was attended by about seventy-five teachers from twenty-three colleges. The program was varied and well considered. There was no lack of interest in any of the subjects discussed, but every one felt exhausted when the meeting was over, because of the close application. With a program committee headed by D. M. Cattell and with G. E. Hunt as executive officer, there was little time lost or misspent. The variety of subjects discussed was such that each found some subject in which he was particularly interested, and we have no doubt that improved methods of teaching will result from the conference of those engaged in teaching, from listening to the well considered theories and experiences of each other. The changing of the name of this organization and the broadening of its work has undoubtedly been an important factor in inlisting the interest and enthusiasm of all teachers in its work. Boston, Baltimore and Washington were not represented in this session, as it was hoped they might be; all other parts of the country New Orleans, San-Francisco, Minneapolis and intermediate cities were well represented. The next meeting will be held at Chicago, and with Dr. H. J. Goslee as president, it will doubtless be the banner meeting. We were about to suggest to the program committee, but we remember that the program is already made up, and ought to be good after a full year of time in which to consider it.

On account of the meeting in Stockholm of the International Dental Federation at a time which makes it impracticable for those wishing to attend the meeting of the National Association of Dental Faculties, to attend both, it is proposed to postpone the meeting of the N. A. D. F. to the Christmas vacation period. A petition to this effect is being circulated for signatures, and will be presented to the executive committee at an early date; the probabilities are that it will prevail. If it should, we believe the meeting of the National Dental Association this year will be attended with more satisfaction, at least by college men.

A bill has been introduced in the United States Senate providing for the attachment of dental surgeons to the medical corps of the navy, to serve the officers, enlisted men and boys in schools and training, in the proportion of one dentist to every one thousand engaged in the service or authorized by law. There are to be three grades in rank; assistant surgeon, past assistant surgeon and surgeon. Moral and professional character as well as educational standing are requisite. The selection of these officers is to be placed in charge of a qualified officer with the rank of surgeon, who shall be appointed because of his peculiar fitness for such office. It is the hope that the bill may soon become a law and every well-wisher is urged to inform his congressman of the value and importance of such appointments.

Dr. A. W. Harlan, for so many years the able and versatile editor of the *Dental Review*, has retired from editorial work and is succeeded by Dr. C. N. Johnson.

We do not know why Dr. Harlan has seen fit to withdraw from a work which he has so honorably and acceptably done for so many years. Perhaps, like the editor of the late *Indiana Dental Journal*, he is "tired." But whatever the reason, the profession of the middle West is greatly indebted to him for placing on permanent record the best thought of the profession, not only of the West, but for the entire professional world. Much of the best writing done in dental literature during the past fifteen years has come within the sphere of the *Dental Review*, and by the enterprise and acuteness of Dr. Harlan the readers of this journal have had it at first hands. Dr. Johnson will be welcomed by the *Review's* readers, and his capacity for graceful writing will no doubt be one of the characteristic features of this journal. While regretting the retirement of Dr. Harlan, we welcome most cordially Dr. Johnson, and trust his conduct of the *Review* may be as successful as his predecessor's.

---

We have just learned that the January issue of the *Ohio Dental Journal* was destroyed by fire, and it will have to be entirely reproduced. We are also informed that this journal, for the second time in its history, will change its name, to the *Dental Summary*. We presume the editor and publishers do this advisedly, but we can not but think it a mistake. The old name of *Ohio Journal of Dental Science* had a greater significance than the present one, and the change now proposed seems like a retrograde movement, as it doubtless contemplates epitomized reprints, which are generally lacking in value, to say nothing of the loss of personality of contributors, and so of interest.

